# Isolation, Serovar Identification, and Antimicrobial Susceptibility of *Avibacterium* *paragallinarum* from Chickens in China from 2019 to 2020

**DOI:** 10.3390/vetsci9010027

**Published:** 2022-01-12

**Authors:** Mengjiao Guo, Xiufang Chen, Hao Zhang, Donghui Liu, Yantao Wu, Xiaorong Zhang

**Affiliations:** 1 Jiangsu Co-Innovation Center for Prevention and Control of Important Animal Infectious Diseases and Zoonoses, College of Veterinary Medicine, Yangzhou University, Yangzhou 225009, China; guomj@yzu.edu.cn (M.G.); MZ120191068@stu.yzu.edu.cn (X.C.); MZ120201477@stu.yzu.edu.cn (H.Z.); MX120190754@stu.yzu.edu.cn (D.L.); ytwu@yzu.edu.cn (Y.W.); 2 Joint International Research Laboratory of Agriculture & Agri-Product Safety, Yangzhou University (JIRLAAPS), Yangzhou 225009, China

**Keywords:** *Avibacterium paragallinarum*, infectious coryza, antimicrobial susceptibility, minimum inhibitory concentration

## Abstract

Infectious coryza is an acute infectious respiratory disease in chickens that is caused by *Avibacterium paragallinarum* (*A. paragallinarum*). Infectious coryza has major economic effects due to decreased egg production in growing birds and slowed growth in broilers. In this study, we isolated and identified 40 strains of *A. paragallinarum* from chickens that showed typical clinical signs of coryza in part of China from 2019 to 2020. Using a hemagglutination-inhibition test, 11 isolates were identified as serovar A, 10 isolates were identified as serovar B, and 19 isolates were identified as serovar C. Antimicrobial sensitivity tests showed that high minimum inhibitory concentration (MIC) values were encountered for compounds sulfamethoxine sodium and oxytetracycline hydrochloride. Especially, of the 40 *A. paragallinarum* isolates, 30% had an MIC value of compound sulfamethoxine sodium of 64 μg/mL, 10% of 128 μg/mL, and 15% of 256 μg/mL. For oxytetracycline hydrochloride, 85% of isolates showed MIC values of 64 μg/mL or more. Excitingly, the MIC values of β-lactamase (amoxicillin, ampicillin, and ceftiofur) were low, with 77.5%, 70%, and 92.5% of isolates having an MIC value of ≤1 μg/mL, respectively. Our results may provide a reference for the treatment of infectious coryza.

## 1. Introduction

Infectious coryza (IC) is an acute infectious respiratory disease in chickens caused by a bacterium of the Pasteurellaceae family, *Avibacterium paragallinarum* (*A. paragallinarum*) [1]. The most prominent character of IC is acute inflammation of the upper respiratory tract, with facial swelling, nasal discharge, and conjunctivitis. IC occurs worldwide and causes significant economic losses due to growth retardation in growing birds and marked drops in egg production in layers. In addition, the control of IC requires intense vaccinations [2]. Stress is an important factor to be considered in the IC occurrence. In field scenarios and experimental infections, coinfection of *A. paragallinarum* with other bacteria and viruses increases clinical signs and pathologic lesions [3,4,5]. In brief, high mortality and airsacculitis are the important issues of IC in broilers [6]. In the past decade, IC has been relatively well controlled in poultry. However, it occurred in many countries, such as China, USA, Indonesia, Great Britain and India, in recent years [7,8,9,10,11]. In China, IC outbreaks have happened in Beijing, Shandong, and Anhui province since 2012. Therefore, the disease prevention measures to control IC need to be improved.

*A. paragallinarum* was serotyped into three serovars (A, B, and C) by hemagglutination inhibition (HI) tests according to the Page scheme [12]. The Kume scheme recognizes the same serogroups as the Page scheme (A1–A4, B–1, and C1–C4) [13,14]. The Kume scheme is also based on HI tests, but it is only used in a few laboratories because of its high technical requirements. The three Page serovars can be isolated from chickens all over the world and the prevalence of serotypes varies from country to country. In China, all three serovars have been reported. Serovar A was first reported in 1987, serovar C in 1995, and serovar B in 2003 [7,15]. Page or Kume serogroups are generally considered to represent three different immunovars [16]. There is no cross-protection among different serovars and the cross-protection within Page serovar B is not universal [17]. There is generally good cross-protection between the four serotypes A. Some of the four serotypes C have partial cross-protection [18]. Therefore, the identification of epidemic Page serovars provides the theoretical support for the selection of an appropriate vaccine.

Strict biosecurity and vaccination are the most important measures to prevent and control IC. In addition to vaccination, it is important to choose appropriate antimicrobial agents to treat and control IC. Many antimicrobial agents have been used, but many of them can only reduce the severity of the disease and cannot completely eliminate *A. paragallinarum* in chickens [11]. Once chickens experience adverse factors, IC easily recurs. Furthermore, if repeated treatment is used, there is a higher risk of the development of antimicrobial resistance to some antimicrobial agents. An increase in the resistance to antimicrobial drugs by *A. paragallinarum* has been reported [19,20,21,22]. However, the information of antimicrobial susceptibility of *A. paragallinarum* in China is scarce. This study aims to isolate and identify *A. paragallinarum* from chickens in several modern, intensive, large chicken farms with typical symptoms of facial swelling and nasal discharge from 2019 to 2020. The commonly used antimicrobial agents erythromycin, tetracycline derivatives, and sulfonamides were not effective in these farms. To provide appropriate treatment for IC, the antimicrobial sensitivity tests for *A. paragallinarum* are of great importance.

## 2. Materials and Methods

### 2.1. Sampling

The typical clinical signs of IC appeared in different chicken farms located in Jiangsu, Hebei, and Ningxia Hui Autonomous Region of China from 2019 to 2020. The chickens showed respiratory symptoms, tears, facial edema, and decreased egg production by more than 50%. In this study, the clinical samples were obtained from 12 modern, intensive, large chicken farms with IC outbreaks, which were submitted to our laboratory for post mortem examination. One hundred and eighty-two samples were collected from the nasal and infraorbital sinuses of chickens with clinical facial edema and discharge. Most isolates were from commercial chicken production systems. Farms located in Hebei and Ningxia Hui Autonomous Region raised layer parent stock. The breed of chickens included Hy-line brown, Jing Brown No. 1, and Nongda 3. We did not collect samples of chicken flocks regularly throughout the year to conduct a systematic epidemiological survey. However, as long as IC occurred in these chicken farms, we collected samples for *A. paragallinarum* isolation and identification. Because the occurrence of IC was mainly concentrated in the change of season (from April to July and from October to November), the samples also concentrated in these times. In addition, due to the influence of Corona Virus Disease 2019, only samples for the second half of the year were collected in 2020. The origin of the samples and isolates is shown in Table 1.

### 2.2. A. paragallinarum Isolation

The samples were cultured on chocolate agar or trypticase soy agar supplemented with 10% fetal bovine serum and 0.0025% reduced nicotinamide adenine dinucleotide at 37 °C under 5% CO_2_ for 24–48 h. One suspected colony with typical *A. paragallinarum* morphology was selected and further streaked on chocolate agar for purification. Afterward, the plates were incubated at 37 °C in a 5% CO_2_ incubator for 24–48 h. The suspected colonies were grown in trypticase soy broth supplemented with 10% fetal bovine serum and 0.0025% reduced nicotinamide adenine dinucleotide at 37 °C for 16 h. The pure culture was identified by Gram staining and classical biochemical methods. Bacterial DNA was extracted using a TIANamp Bacteria DNA kit (Transgen Biotech Co., Ltd., Beijing, China), according to the manufacturer’s instructions. To determine if the isolates were *A. paragallinarum*, all of them were identified using primers of HPG-2 F (TGAGGGTAGTCTTGCACGCGAAT) and HPG-2 R (CAAGGTATCGATCGTCTCTCTACT), which are specific to *A. paragallinarum* [23]. All the *A. paragallinarum* isolates were kept at −70 °C until they were used.

### 2.3. Serotyping

All *A. paragallinarum* isolates were serotyped with specific antisera according to the Page scheme. The reference strains of *A. paragallinarum* 221 (serovar A), Spross (serovar B), and H-18 (serovar C) were purchased from China Veterinary Culture Collection Center (Beijing, China) and were used as control. Antisera for the hemagglutination-inhibition (HI) test were obtained as reported in a previous study [24]. The isolates were grown in trypticase soy broth supplemented with 10% fetal bovine serum and 0.0025% reduced nicotinamide adenine dinucleotide at 37 °C for 16 h. After incubation, the isolates were centrifuged, washed with phosphate-buffered saline, and treated with potassium thiocyanate for 2 h at 4 °C. The bacteria were sonicated, centrifuged, washed, and resuspended in phosphate-buffered saline as antigens. The glutaraldehyde-fixed chicken erythrocytes were used for the HI test. The serotypes of the isolates corresponded to the antiserum with the highest HI titer.

### 2.4. Antimicrobial Sensitivity Test

All isolates and reference strains (221, Spross, and H-18) were tested by the minimum inhibitory concentration (MIC) with the most commonly used antimicrobial agents. The modified broth microdilution method referred to the performance standards for antimicrobial disk and dilution susceptibility tests for bacteria isolated from animals developed in the Clinical and Laboratory Standards Institute (CLSI, 2013). Isolates and reference strains were grown in trypticase soy broth supplemented with 10% fetal bovine serum and 0.0025% reduced nicotinamide adenine dinucleotide at 37 °C for 16 h. The *A. paragallinarum* culture was diluted in trypticase soy broth medium to 10^7^ cfu/mL. The 96-well microdilution plates were used for the MIC test. The antimicrobial agents were double-diluted to 0.0625–256 μg/mL with 100 μL trypticase soy broth medium. Then, the 100 μL diluted *A. paragallinarum* culture was inoculated into each well. The *A. paragallinarum* culture and antimicrobial agents were used as negative control and antibiotic control, respectively. Then, the plates were incubated at 37 °C for 20 h. The MIC values were determined by a photometer. Valnemulin hydrochloride (VA), compound sulfamethoxine sodium (COSMMS), doxycycline hydrochloride (DO), oxytetracycline hydrochloride (OT), amoxicillin (AMX), florfenicol (FFC), lincomycin/spectinomycin (LS), gentamycin sulfate (CN), enrofloxacin (ENR), ampicillin (AMP), tylvalosin tartrate (TAT), and ceftiofur (CTF) were used for this study. Three independent experiments were conducted.

## 3. Results

### 3.1. Isolation and Serotyping of A. paragallinarum

Dew-like colonies were identified on chocolate agar or trypticase soy agar supplemented with 10% fetal bovine serum and 0.0025% reduced nicotinamide adenine dinucleotide. After biochemical and PCR testing, a total of 40 *A. paragallinarum* strains were isolated and identified from clinical samples of suspected IC disease. The isolation rate of *A. paragallinarum* was 22%. Combined with the incidence information provided by the chicken farms, IC occurs frequently in the change of season (from April to July and from October to November). Chickens were infected mainly in the laying period, as well as in the brooding period, at 30–40 days old. The results of the HI test showed that 11 isolates were identified as serovar A, 10 isolates were identified as serovar B, and 19 isolates were identified as serovar C. In most chicken farms, only one serotype was prevalent in the process of an outbreak. However, two serotypes were prevalent during an outbreak in farms G, I, and J. Three serotypes were prevalent during an outbreak in farm F. Serovar A was prevalent in April 2019, but serovar C was prevalent in October 2020 in farm B.

### 3.2. Antimicrobial Susceptibility Testing

**Figure 1:**
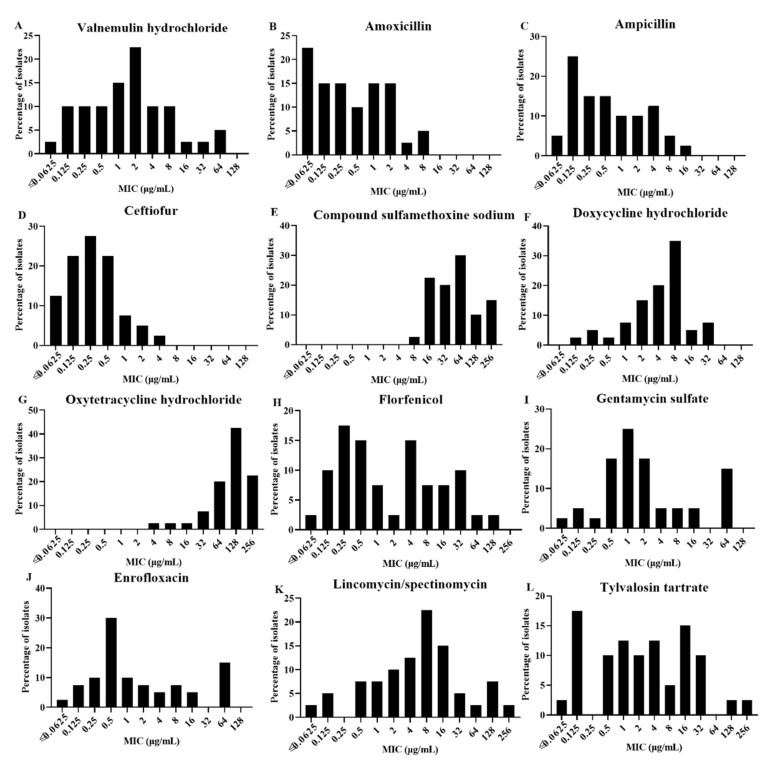
Distribution of minimum inhibitory concentration values of antimicrobial agents tested: (**A**) alnemulin hydrochloride, (**B**) amoxicillin, (**C**) ampicillin, (**D**) ceftiofur, (**E**) compound sulfamethoxine sodium, (**F**) doxycycline hydrochloride, (**G**) oxytetracycline hydrochloride, (**H**) florfenicol, (**I**) gentamycin sulfate, (**J**) enrofloxacin, (**K**) lincomycin/spectinomycin, and (**L**) tylvalosin tartrate.

Table 2 shows the MICs of 12 antimicrobial agents against 40 *A. paragallinarum* isolates and reference strains (221, Spross, and H-18). The MIC values ranged from ≤0.0625 to 64 μg/mL for VA. As shown in Figure 1A, 10% of isolates showed MIC values of 16 μg/mL or more for VA. The MIC values of other β-lactamase (AMX, AMP, and CTF) were low (Figure 1B–D), while high MIC values were encountered for COSMMS and OT. Especially, the MIC values ranged from ≤0.0625 to 4 μg/mL for CTF and 92.5% of isolates showed MIC ≤ 1 μg/mL. Of the 40 *A. paragallinarum* isolates, 2.5% had an MIC value of COSMMS of 8 μg/mL, 22.5% of 16 μg/mL, 20% of 32 μg/mL, 30% of 64 μg/mL, 10% of 128 μg/mL, and 15% of 256 μg/mL (Figure 1E). MIC values ranged from ≤0.125 to 32 μg/mL for DO, with 12.5% of isolates showing MIC values of 16 μg/mL or more (Figure 1F), while 85% of isolates showed MIC values of 64 μg/mL or more for OT (Figure 1G). About 20% of isolates showed MIC values of 16 μg/mL or more for FFC, CN, and ENR (Figure 1H–J). The *A. paragallinarum* isolates displayed a wide variance in MIC values for LS with a range from ≤0.0625 to 256 µg/mL and the same was observed for TAT, a macrolide antibiotic. Of the 40 isolates, 32.5% and 30% of isolates showed MIC values of 16 μg/mL or more for LS and TAT, respectively (Figure 1K,L).

## 4. Discussion

IC has worldwide economic significance and leads to poor growth performance of broilers, as well as decreased egg production among layers. IC can be found all over the world, but its pathogen, *A. paragallinarum*, is difficult to isolate due to the use of antibiotics in feed [25]. Moreover, *A. paragallinarum* must be isolated in the acute infection stage. It is a slow-growing and fastidious bacterium and most strains need V-(nicotinamide adenine dinucleotide) factor for growth in vitro. In addition, *A. paragallinarum* is easily covered up and overgrown by other *Pasteurellaceae* bacteria in the process of isolation and culture [9]. Conventionally, IC can be preliminarily diagnosed according to the rapid spread of the disease and coryza symptoms. The diagnosis is confirmed by isolates with satellite growth in blood agar plates [1].

Since 2020, all forms of growth-promoting antibiotics except traditional Chinese medicines have been forbidden to be used as feed additives in China. Although they can be used for treatment, many antibiotics are banned during the laying period or have a strict rest period. With the restricted use of antibiotics, the morbidity and isolation rate of IC has gradually increased in China. In China, *A. paragallinarum* serovars A, B and C have been reported. Serovars A and C were the major serotypes causing outbreaks of IC, until serovar B appeared in 2003. After that, *A. paragallinarum* serovar B was detected in Beijing and Tianjin. Over the years, the incidence of Page serovar B infection has significantly increased in the world, including China, Brazil, Egypt, Mexico, South Africa, Germany, and the United States [1,26,27]. In recent years, 28 *A. paragallinarum* serovar B isolates were isolated from chickens in the Shandong, Liaoning, Hebei, Beijing, Anhui, Sichuan, Jiangsu, and Guangdong provinces [28]. It has been reported that *A. paragallinarum* serovar B isolates are as pathogenic as serovars A and C isolates [7]. In this study, 40 isolates were obtained from 12 modern, intensive, large chicken farms with IC outbreaks and confirmed by HPG-2 PCR. Page serovars A, B, and C were all isolated and 10 isolates were identified as serovar B. In most chicken farms, only one serotype was prevalent during an outbreak. However, two serotypes occurred during an outbreak in farms G, I, and J. Serovars A, B, and C broke out in farm F at the same time. Our results suggested that the immunization of a trivalent vaccine is necessary. A previous study has demonstrated that only partial cross-protection has been seen among serovar B isolates, although there is only one Kume serovar B (B-1). IC broke out even in chickens immunized with bivalent (A + C) or trivalent (A + B + C) inactivated vaccines, which indicated that some cases were possibly related to vaccine failure.

*Hmtp210* of *A. paragallinarum* encodes a 210 kDa outer membrane protein [29]. Previous research proved that *Hmtp210* plays a key role in the pathogenicity of *A. paragallinarum*, which has the function of hemagglutination, cell adhesion, and biofilm formation activity [30]. The hypervariable region of *Hmtp210* located at about 1100–1600 aa is considered to be the most antigenic region of *Hmtp210*. The recombinant vaccines for the hypervariable region of *Hmtp210* protect chickens against challenge with *A. paragallinarum* [31,32]. It has been reported that *Hmtp210* is an important protective antigen and a candidate for serotyping [33]. A multiplex PCR and PCR-RFLP method using the hypervariable region of *Hmtp210* was developed to identify the serovar of *A. paragallinarum* [34]. However, we also performed this multiplex PCR and demonstrated that this method cannot be used for serotyping. Our result is consisted with Wang et al.’s [35]. The HI test is one of the most widely used serological tests. It is usually used to detect antibody titers and serotype, followed by epidemiological studies to assess the prevalence of IC. The Page scheme is still the most commonly used and effective serotype method. Therefore, the HI test was used to serotype the *A. paragallinarum* isolates in this study.

The treatment of IC has not been widely studied. However, the use of some antimicrobial agents has been reported, especially sulfonamides [36,37]. Recently, *A. paragallinarum* isolates have shown resistance to many antimicrobial agents, such as streptomycin, sulfonamides, and OT [20,22]. In present study, high MIC values were encountered for COSMMS and OT. Of the 40 *A. paragallinarum* isolates, 30% had an MIC value of COSMMS of 64 μg/mL, 10% of 128 μg/mL, and 15% of 256 μg/mL. Our results showed that 95% of isolates were characterized by high MIC values of OT (≥16 μg/mL), while 12.5% of isolates showed MIC values of ≥16 μg/mL for DO, the other tetracycline. In previous research, 72.2% of *A. paragallinarum* isolates had an MIC value of ≥16 μg/mL for OT. For DO, MIC values of ≥16 μg/mL were detected in 66.7% of Thailand isolates [38]. Low MICs of AMP and penicillin were found in Australian field isolates, having MIC values of ≤0.5 and ≤1 μg/mL [39]. The MIC values of β-lactamase (VA, AMX, AMP, and CTF) were tested in this study; in total, 47.5%, 77.5%, 70%, and 92.5% of isolates had an MIC value of ≤1 μg/mL, respectively. Similar to our results, 83.3% of Thailand isolates were characterized by an MIC ≤ 1 μg/mL for AMP. For all 44 isolates in Dutch isolates, the MIC values of AMP were ≤ 1 μg/mL [19]. Taiwan isolates differed from ours, with only 27.8% isolates having MIC values of AMP ≤ 1 μg/mL [40]. For ENR, 50.0% Thailand isolates’ MIC values were ≥ 4 μg/mL [38], while all Dutch isolates’ MIC values were ≤ 2 μg/mL [19]. In this study, 32.5% isolates’ MIC values were ≥ 4 μg/mL. The high MIC values of CN, LS, OT, and TAT, matches the results of previous research studies using agar diffusion [21,22,41].

High MIC values of COSMMS and OT were observed in this study. Since these antimicrobial agents are commonly used in the treatment of IC, the results also suggest that they may not be effective in future treatments in China. Thus, antimicrobial sensitivity tests need be carried out for the selection of effective antimicrobial agents. However, excitingly, we found that the MIC values of β-lactamase were low, especially CTF and AMX. In the following treatment, we recommended AMX and CTF for IC treatment and obtained a good effect in chicken farms.

## 5. Conclusions

In this study, 182 samples were collected from the nasal and infraorbital sinuses of chickens with clinical facial edema and discharge in 12 modern, intensive, large chicken farms from 2019 to 2020. In total, 40 *A. paragallinarum* strains were isolated and identified; specifically, 11 isolates were identified as serovar A, 10 isolates were identified as serovar B, and 19 isolates were identified as serovar C. In most chicken farms, only one serotype was prevalent during an outbreak. However, serovars A, B, and C broke out in farm F at the same time. Our results suggest that the immunization of a trivalent vaccine is necessary. The antimicrobial susceptibility was investigated using an MIC test. The *A. paragallinarum* isolates displayed a wide variance in MICs for LS, with a range from ≤0.0625 to 256 µg/mL, and the same was observed for TAT. The MIC values ranged from ≤0.0625 to 64 μg/mL for VA. The MIC values of β-lactamase (AMX, AMP, and CTF) were low, while high MIC values were observed for COSMMS and OT. Especially, the MIC values ranged from ≤0.0625 to 4 μg/mL for CTF, with 92.5% of isolates showing an MIC ≤ 1 μg/mL. For COSMMS, 10% isolates had an MIC value of 128 μg/mL and 15% of 256 μg/mL. β-lactamases AMX and CTF were effective in the treatment of IC and could be used as a reference treatment strategy for the disease. The information provided by the isolation, serovar identification, and antimicrobial susceptibility of *A. paragallinarum* will allow researchers to design a more effective use of antimicrobial agents or other methods of controlling IC.

## Figures and Tables

**Table 1:** Origin of *Avibacterium paragallinarum* isolates.

Farm ID	Isolates	Origin	Production Type/Breed of Host	Size of Flocks	Husbandry Systems	Age	Time	Serovar
A	2019/JS03	Jiangsu	Hy-line brown, layer	2,000,000	H-type stacked-cage	326	2019/04	A
A	2019/JS07	Jiangsu	Hy-line brown, layer	2,000,000	H-type stacked-cage	264	2019/04	A
B	2019/JS08	Jiangsu	Hy-line brown, layer	1,200,000	H-type stacked-cage	241	2019/04	A
B	2019/JS15	Jiangsu	Hy-line brown, layer	1,200,000	H-type stacked-cage	316	2019/04	A
C	2019/JS28	Jiangsu	Hy-line brown, layer	500,000	H-type stacked-cage	207	2019/04	A
D	2019/JS31	Jiangsu	Hy-line brown, layer	600,000	H-type stacked-cage	255	2019/07	B
D	2019/JS33	Jiangsu	Hy-line brown, layer	600,000	H-type stacked-cage	212	2019/07	B
A	2019/JS34	Jiangsu	Hy-line brown, layer	2,000,000	H-type stacked-cage	278	2019/07	B
A	2019/JS35	Jiangsu	Hy-line brown, layer	2,000,000	H-type stacked-cage	278	2019/07	B
E	2019/JS36	Jiangsu	Hy-line brown, layer	1,000,000	H-type stacked-cage	322	2019/07	B
E	2019/JS37	Jiangsu	Hy-line brown, layer	1,000,000	H-type stacked-cage	318	2019/07	B
E	2019/JS38	Jiangsu	Hy-line brown, layer	1,000,000	H-type stacked-cage	42	2019/07	B
F	2019/JS39	Jiangsu	Jing Brown No. 1, layer	300,000	H-type stacked-cage	78	2019/07	B
F	2019/JS40	Jiangsu	Jing Brown No. 1, layer	300,000	H-type stacked-cage	133	2019/07	B
F	2019/JS42	Jiangsu	Jing Brown No. 1, layer	300,000	H-type stacked-cage	311	2019/07	A
F	2019/JS44	Jiangsu	Jing Brown No. 1, layer	300,000	H-type stacked-cage	332	2019/07	C
G	2019/JS45	Jiangsu	Nongda 3, layer	600,000	H-type stacked-cage	276	2019/07	A
G	2019/JS46	Jiangsu	Nongda 3, layer	600,000	H-type stacked-cage	306	2019/07	C
H	2019/ NX56	Ningxia	Hy-line brown, layer parent stock	300,000	Net-rearing	333	2019/10	C
H	2019/ NX57	Ningxia	Hy-line brown, layer parent stock	300,000	Net-rearing	324	2019/10	C
H	2019/ NX58	Ningxia	Hy-line brown, layer parent stock	300,000	Net-rearing	312	2019/10	C
I	2019/ HB63	Hebei	Hy-line brown, layer parent stock	300,000	Natural mating cage	103	2019/11	A
I	2019/ HB64	Hebei	Hy-line brown, layer parent stock	300,000	Natural mating cage	128	2019/11	A
I	2019/ HB65	Hebei	Hy-line brown, layer parent stock	300,000	Natural mating cage	164	2019/11	A
I	2019/ HB68	Hebei	Hy-line brown, layer parent stock	300,000	Natural mating cage	164	2019/11	B
J	2020/JS69	Jiangsu	Hy-line brown, layer	100,000	H-type stacked-cage	273	2020/10	A
J	2020/JS70	Jiangsu	Hy-line brown, layer	100,000	H-type stacked-cage	266	2020/10	C
F	2020/JS71	Jiangsu	Jing Brown No. 1, layer	300,000	H-type stacked-cage	213	2020/10	C
F	2020/JS72	Jiangsu	Jing Brown No. 1, layer	300,000	H-type stacked-cage	298	2020/10	C
F	2020/JS73	Jiangsu	Jing Brown No. 1, layer	300,000	H-type stacked-cage	246	2020/10	C
B	2020/JS74	Jiangsu	Hy-line brown, layer	1,200,000	H-type stacked-cage	323	2020/10	C
B	2020/JS75	Jiangsu	Hy-line brown, layer	1,200,000	H-type stacked-cage	302	2020/10	C
B	2020/JS76	Jiangsu	Hy-line brown, layer	1,200,000	H-type stacked-cage	195	2020/10	C
G	2020/JS77	Jiangsu	Nongda 3, layer	600,000	H-type stacked-cage	311	2020/11	C
F	2020/JS78	Jiangsu	Hy-line brown, layer	300,000	H-type stacked-cage	204	2020/11	C
G	2020/JS79	Jiangsu	Nongda 3, layer	600,000	H-type stacked-cage	362	2020/11	C
G	2020/JS80	Jiangsu	Nongda 3, layer	600,000	H-type stacked-cage	188	2020/11	C
K	2020/JS81	Jiangsu	Hy-line brown, layer	200,000	H-type stacked-cage	76	2020/11	C
K	2020/JS82	Jiangsu	Hy-line brown, layer	200,000	H-type stacked-cage	34	2020/11	C
L	2020/JS83	Jiangsu	Hy-line brown, layer	300,000	H-type stacked-cage	57	2020/11	C

**Table 2:** Minimum inhibitory concentration of *A. paragallinarum* isolates.

Strains	MIC (μg/mL)											
	VA	AMX	CTF	AMP	COSMMS	DO	OT	FFC	CN	ENR	LS	TAT
221	0.125	0.125	0.25	≤0.0625	≤0.0625	2	1	1	0.125	0.5	32	0.25
Spross	≤0.0625	≤0.0625	0.25	0.5	≤0.0625	≤0.0625	0.125	2	0.25	0.125	0.5	0.5
H-18	0.25	0.25	0.25	0.25	≤0.0625	2	16	0.5	0.125	1	16	0.5
2019/JS03	1	≤0.0625	0.25	0.25	16	2	32	8	0.5	0.25	4	2
2019/JS07	2	0.25	0.5	2	32	4	64	8	2	1	16	16
2019/JS08	1	0.5	≤0.0625	0.25	32	4	32	4	0.5	0.25	16	8
2019/JS15	1	0.25	0.25	0.5	32	8	64	1	1	2	8	16
2019/JS28	32	8	2	4	64	8	32	16	16	64	16	16
2019/JS42	64	1	0.5	2	256	32	256	64	64	64	256	256
2019/JS45	64	1	0.5	4	256	32	128	128	64	64	128	128
2019/HB63	4	4	0.25	8	256	4	64	32	64	8	32	1
2019/HB64	2	1	0.5	1	128	16	256	0.5	64	64	16	32
2019/HB65	2	0.25	0.25	1	64	32	256	4	64	64	16	32
2020/JS69	0.25	0.125	0.125	8	32	1	64	0.25	0.125	0.125	0.125	0.125
2019/JS31	2	0.5	1	0.5	128	8	128	4	1	8	128	32
2019/JS33	8	2	1	0.5	256	4	128	8	1	8	128	16
2019/JS34	16	0.5	0.25	0.5	256	8	256	4	1	1	64	32
2019/JS35	0.5	1	0.125	0.125	32	8	128	1	1	4	4	2
2019/JS36	0.0625	0.0625	0.125	0.125	128	16	128	32	1	0.5	8	2
2019/JS37	2	2	2	4	256	8	256	32	8	16	32	8
2019/JS38	1	1	0.5	1	128	8	128	16	1	2	8	4
2019/JS39	4	2	0.5	0.25	64	8	128	32	1	4	2	4
2019/JS40	8	0.25	1	16	64	8	64	16	1	1	16	4
2019/HB68	2	0.5	0.125	0.125	64	4	64	0.5	1	0.5	8	4
2019/JS44	0.125	≤0.0625	0.25	0.0625	32	2	64	0.25	0.5	0.5	2	0.5
2019/JS46	0.125	0.125	4	0.0625	16	1	64	0.125	64	64	4	1
2019/NX56	2	2	0.5	2	8	1	4	0.25	0.5	1	0.125	0.5
2019/NX57	0.5	0.25	0.125	0.125	16	0.5	8	0.5	0.25	16	4	1
2019/NX58	0.5	0.0625	0.0625	0.5	16	4	16	4	0.0625	0.125	2	0.0625
2020/JS70	2	1	0.25	0.5	32	8	128	2	0.125	0.0625	0.0625	0.5
2020/JS71	8	2	0.125	0.25	64	8	128	0.5	2	0.5	8	16
2020/JS72	1	0.125	0.5	2	16	0.125	128	0.25	0.5	0.5	1	0.125
2020/JS73	8	0.0625	0.0625	0.25	64	8	128	1	2	0.5	8	16
2020/JS74	4	2	0.25	4	16	2	256	0.25	4	0.5	8	1
2020/JS75	0.125	0.0625	0.5	0.125	16	0.25	256	4	0.5	0.5	0.5	0.5
2020/JS76	2	0.25	0.25	4	32	4	128	0.5	4	0.5	8	4
2020/JS77	1	0.0625	0.0625	0.125	16	0.25	128	0.25	2	0.5	0.5	1
2020/JS78	0.25	0.0625	0.25	0.25	64	8	128	0.125	0.5	0.5	4	0.125
2020/JS79	0.125	0.125	0.25	1	64	2	128	0.125	8	0.125	2	0.125
2020/JS80	0.5	0.125	0.125	0.125	64	4	256	0.0625	2	0.25	1	2
2020/JS81	0.25	0.0625	0.0625	0.125	16	2	256	0.5	2	0.25	0.5	0.125
2020/JS82	0.25	0.125	0.125	0.125	64	8	128	0.25	2	0.5	8	0.125
2020/JS83	4	8	0.125	0.125	64	2	128	0.125	16	2	1	0.125

## Data Availability

The data presented in this study are available in the article.
